# Author Correction: SARS-CoV-2 seroprevalence in healthcare workers at a frontline hospital in Tokyo

**DOI:** 10.1038/s41598-021-95498-2

**Published:** 2021-08-02

**Authors:** Hiroshi Fukuda, Kuniaki Seyama, Kanami Ito, Tomohiko Ai, Shuko Nojiri, Satoshi Hori, Mitsuru Wakita, Kaori Saito, Yuka Shida, Rie Nagura, Mayu Hasegawa, Chiaki Kanemoto, Mayumi Tokuhara, Katsunobu Okajima, Yukio Yoshikawa, Narimasa Katsuta, Takamasa Yamamoto, Mayumi Idei, Yuki Horiuchi, Kotoko Yamatani, Shigeki Misawa, Toshio Naito, Takashi Miida, Hiroyuki Sato, Nobutaka Hattori, Yoko Tabe, Kazuhisa Takahashi

**Affiliations:** 1 Department of General Medicine, Juntendo University Graduate School of Medicine, Bunkyo, Tokyo Japan; 2 Department of Respiratory Medicine, Juntendo University Graduate School of Medicine, Bunkyo, Tokyo Japan; 3 Department of Safety and Health Promotion, Juntendo University, Bunkyo, Tokyo Japan; 4 Department of Clinical Laboratory Medicine, Juntendo University Graduate School of Medicine, 2-1-1, Hongo, Bunkyo, Tokyo 113-8421 Japan; 5 Medical Technology Innovation Center, Juntendo University, Bunkyo, Tokyo Japan; 6 Infection Control Unit, Juntendo University Hospital, Bunkyo, Tokyo Japan; 7 Department of Infection Control Science, Juntendo University Graduate School of Medicine, Bunkyo, Tokyo Japan; 8 Department of Clinical Laboratory, Juntendo University Hospital, Bunkyo, Tokyo Japan; 9 Department of Neurology, Juntendo University Graduate School of Medicine, Bunkyo, Tokyo Japan; 10 Department of Next Generation Hematology Laboratory Medicine, Juntendo University Graduate School of Medicine, Bunkyo, Tokyo Japan

Correction to: *Scientific Reports* 10.1038/s41598-021-87688-9, published online 16 April 2021

The original version of this Article contained errors in the Affiliations.

All authors were incorrectly affiliated with “Department of Psychiatry, Faculty of Medicine, Juntendo University, Bunkyo, Tokyo, Japan”.

As a result, Affiliations 9-10 were incorrectly listed as Affiliations 10-11.

The original Article has been corrected.

